# Paraneoplastic Amyopathic Dermatomyositis Associated With a Bone and Medullary Location of Breast Adenocarcinoma

**DOI:** 10.7759/cureus.86031

**Published:** 2025-06-15

**Authors:** Fadwa Haboub, Mohamed El Fadli, Othmane Zouiten, Leila Afani, Rhizlane Belbaraka

**Affiliations:** 1 Department of Medical Oncology, Centre Hospitalo-Universitaire Mohammed VI, Marrakesh, MAR; 2 Department of Medical Oncology, Cadi Ayyad University, Marrakesh, MAR

**Keywords:** breast cancer, cdk4/6 inhibitors, erythroderma, medullary location, paraneoplastic amyopathic dermatomyositis

## Abstract

Paraneoplastic amyopathic dermatomyositis (ADM) is a rare connective tissue disease presenting with characteristic dermatomyositis-like skin findings in the absence of muscle involvement. It is associated with malignancies in 15%-30% of cases and may portend a fatal outcome, especially when linked to advanced cancers.

We report the case of a 58-year-old woman who presented with progressive low back pain and erythroderma. Imaging revealed diffuse bone metastases, and bone biopsy confirmed a poorly differentiated adenocarcinoma of breast origin (estrogen receptor, 60%; progesterone receptor, 35%; GATA3 positive). Simultaneously, dermatological evaluation identified periungual erythema and other cutaneous findings consistent with ADM. The patient was started on endocrine therapy with palbociclib and letrozole, along with corticosteroids and hydroxychloroquine, resulting in partial clinical improvement.

However, within six months of diagnosis, she developed severe COVID-19 pneumonia, complicated by pancytopenia and sepsis, and ultimately succumbed to multiorgan failure despite intensive care support.

This case underlines the importance of recognizing paraneoplastic ADM as an initial manifestation of malignancy. A multidisciplinary approach is key, but the prognosis largely depends on the cancer's burden and complications such as treatment-induced immunosuppression.

## Introduction

Paraneoplastic amyopathic dermatomyositis (ADM) is a rare form of dermatomyositis, a connective tissue disease characterized by distinctive skin findings without clinical or laboratory evidence of muscle involvement [1]. Unlike classic dermatomyositis, which presents with both skin and muscle symptoms, ADM manifests with hallmark cutaneous signs such as Gottron’s papules and a heliotrope rash in the absence of muscle weakness or elevated muscle enzymes [1].

A well-established association exists between dermatomyositis and malignancy. Studies have shown that approximately 15%-30% of patients with dermatomyositis have an underlying cancer, either at the time of diagnosis or shortly thereafter. Dermatomyositis can precede, coincide with, or follow the diagnosis of cancer, and virtually all types of malignancies have been reported, including breast, ovarian, lung, and gastrointestinal cancers [2]. In such cases, dermatomyositis is considered a paraneoplastic phenomenon, and the overall prognosis is closely tied to the type and stage of the associated tumor, as well as its response to therapy [2].

In this report, we present a rare case of paraneoplastic ADM associated with metastatic breast adenocarcinoma involving the bone and spinal cord, highlighting the need for thorough malignancy screening in patients presenting with ADM.

## Case presentation

**Figure 1:**
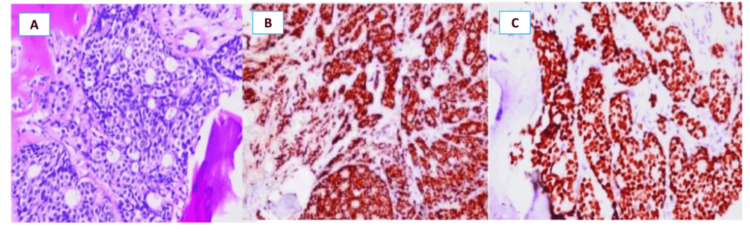
Metastatic adenocarcinoma in the bone marrow. (A) Bone marrow infiltration by tumor cells. Immunohistochemical staining shows nuclear positivity for (B) ER and (C) GATA-binding protein 3 (GATA3), supporting a breast origin ER: estrogen receptor

**Figure 2:**
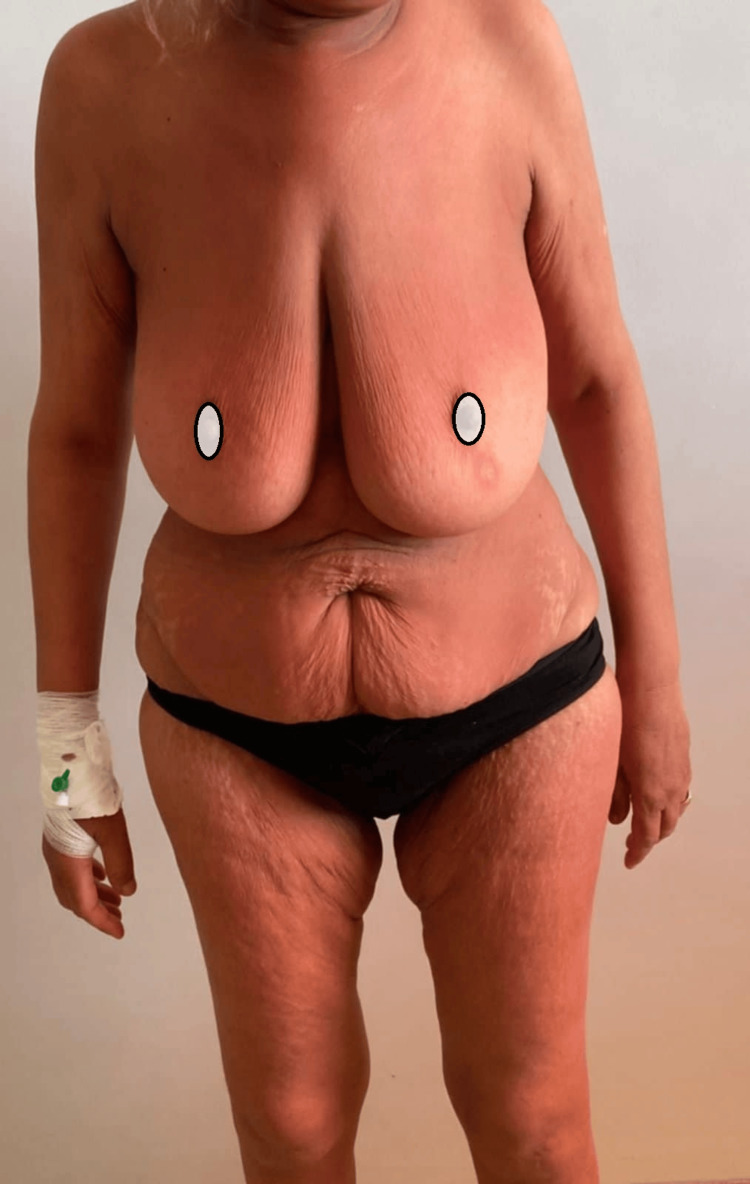
Cutaneous manifestations of dermatomyositis: lilac erythema over the chest and upper trunk, and Gottron's papules over the extensor surfaces of the hands

A 58-year-old woman with a history of high blood pressure was treated with monotherapy. Her history of the disease dates back one year to the onset of low back pain, resistant to analgesic treatment. This was associated with dry erythroderma of the face and back. All of this was occurring in the context of maintaining overall medical health. A lumbar MRI was performed as part of the evaluation for lower back pain, revealing signal abnormalities in the lumbar vertebrae. Subsequent bone scintigraphy demonstrated areas of increased radiotracer uptake in the scapula, scapulohumeral joint, rib cage, and dorsolumbar spine, with intense hyperfixation at L4 and the iliac bones. The (18F)-fluorodeoxyglucose positron-emission tomography/computed tomography (CT) scan revealed hypermetabolic mediastino-hilar lymph nodes with a maximal standardized uptake value (SUV_max_) of 7.2, along with secondary bone involvement affecting both the axial and peripheral skeleton (SUV_max_: 10.6). A bone biopsy revealed a hypoplastic hematopoietic marrow with the presence of suspicious neoplastic cells. Immunohistochemical analysis showed positivity for GATA3, a transcription factor frequently expressed in breast epithelial cells and commonly used as a diagnostic marker for breast cancer metastases. Additionally, the tumor cells were positive for estrogen receptors (ER) at 60% and progesterone receptors at 35%, confirming the hormone-sensitive nature of the malignancy and strongly supporting a breast origin of the poorly differentiated adenocarcinoma infiltrating the marrow (Figure 1). Concerning the erythroderma, collaboration with the dermatology department led to the diagnosis of paraneoplastic ADM, based on the simultaneous onset of cutaneous symptoms with the cancer diagnosis, the presence of characteristic periungual erythema (Figure 2), and elevated lactate dehydrogenase levels (820 IU/L) (Table 1), which may reflect tissue damage or tumor burden. A muscle biopsy from the vastus lateralis showed no abnormalities, supporting the amyopathic form of dermatomyositis.

**Table 1:** Summary of key laboratory findings with corresponding reference ranges

Parameter	Result	Reference range	Units
Lactate dehydrogenase	820	140-280	IU/L
Hemoglobin	5.8	12-16 (female)	g/dL
White blood cell count	920	4,000-11,000	/mm³
Platelet count	30,000	150,000-400,000	/mm³
C-reactive protein	172	<5	mg/L
Procalcitonin	15	<0.5	ng/mL

**Figure 3:**
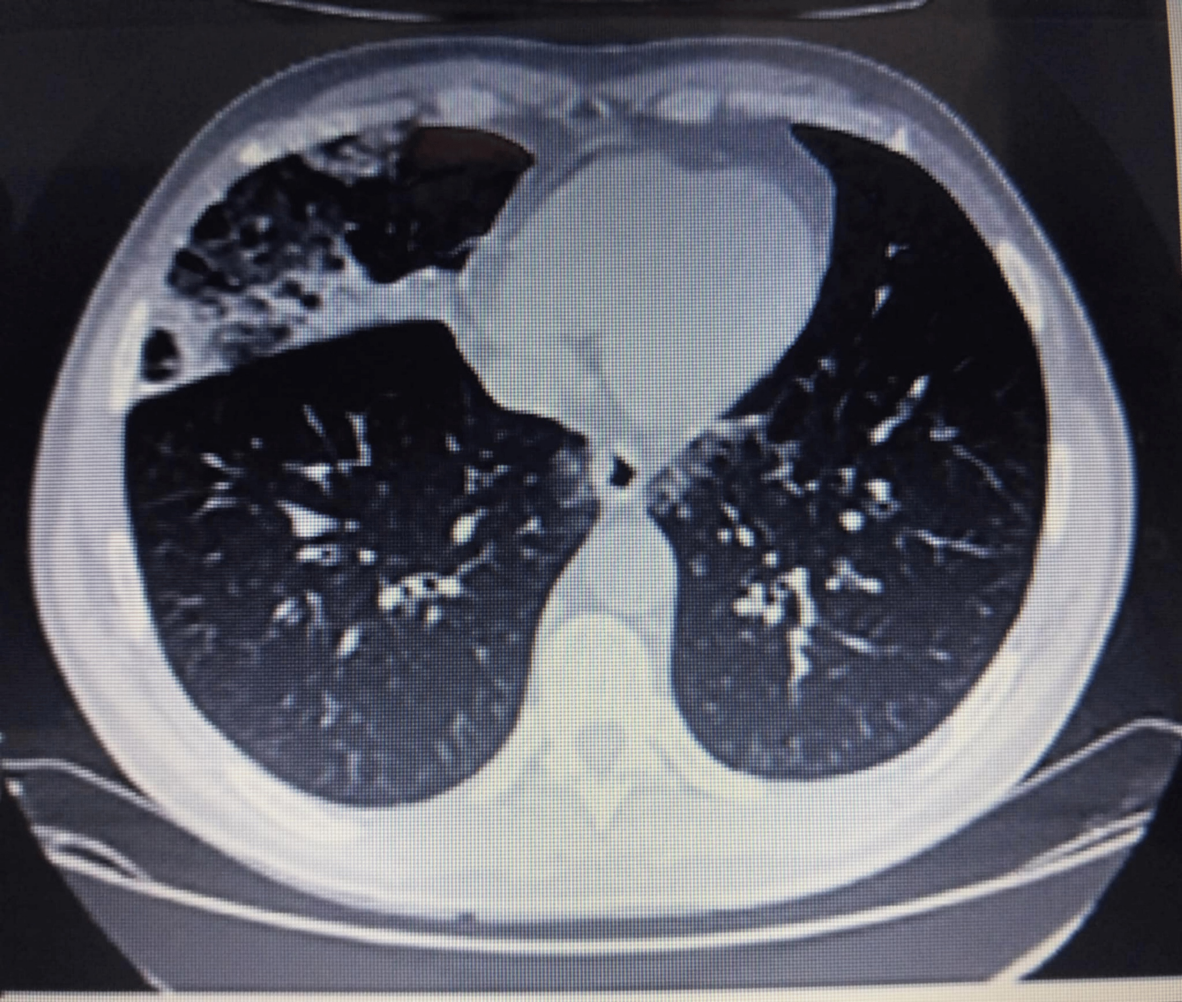
Thoracic CT scan showing bilateral pleural effusion with right-sided predominance, parenchymal consolidations, and ground-glass opacities suggestive of infection CT: computed tomography

Given the hormone receptor-positive profile of the tumor, we initiated a targeted endocrine therapy combining palbociclib, a selective cyclin-dependent kinase (CDK)4/6 inhibitor, with letrozole, an aromatase inhibitor. This regimen is a standard first-line treatment for advanced ER-positive, human epidermal growth factor receptor 2-negative breast cancer in postmenopausal women, aimed at blocking estrogen signaling and inhibiting cell cycle progression to slow tumor growth. With regard to skin involvement, the patient was put on prednisone-type corticosteroid therapy, 60 mg/day, with a synthetic antimalarial (hydroxychloroquine) 400 mg/day. After three months of treatment, we noted a regression of skin lesions, an improvement in lower back pain, and radiological stability. During the sixth cycle of treatment, the patient developed respiratory distress. Biological tests revealed pancytopenia, with a marked reduction in red blood cells, white blood cells, and platelets. The hemoglobin level had dropped to 5.8 g/dL, the white blood cell count to 920/mm³, and the platelet count to 30,000/mm³, suggesting bone marrow suppression. Inflammatory markers were significantly elevated, with a C-reactive protein level of 172 mg/L and a procalcitonin level of 15 ng/mL (Table 1), indicative of a severe infectious process. A thoracic CT scan showed bilateral pleural effusion of moderate size, more prominent on the right, with parenchymal consolidations and ground-glass opacities in the right lung, suggestive of an infectious etiology (Figure 3).

Reverse transcription polymerase chain reaction testing for SARS-CoV-2 RNA was performed, confirming a positive result. The patient was subsequently admitted to the intensive care unit, intubated, and placed on mechanical ventilation, along with a double course of antibiotics. Despite these interventions, the patient unfortunately succumbed after one week of hospitalization.

## Discussion

Paraneoplastic ADM is a distinct subset of dermatomyositis characterized by typical cutaneous findings in the absence of clinically evident muscle involvement. It predominantly affects women, with a reported female-to-male ratio of approximately 2:1 [3,4]. While ADM is relatively frequent in Asian populations, particularly in China, where it represents up to 25% of all dermatomyositis cases [3], it remains rare in Mediterranean regions. It most commonly affects young adults [4], with heliotrope erythema and Gottron's papules being the hallmark dermatological features [3].

Dermatomyositis has been reported in association with various malignancies, regardless of histological subtype, and can precede, coincide with, or follow the diagnosis of cancer [5]. In Europe, the most commonly associated neoplasms include ovarian, breast, lung, colorectal, and prostate cancers, as well as non-Hodgkin lymphoma [6,7]. In women, dermatomyositis has a particularly strong link with gynecologic and breast malignancies, while in men, it is more often associated with bronchopulmonary and digestive cancers, as well as hematologic malignancies [7].

The underlying pathogenesis of paraneoplastic dermatomyositis remains incompletely understood. It is believed to involve dysregulation of both humoral and cellular immunity [5]. The paraneoplastic form, reported in 15-30% of cases, likely results from immune cross-reactivity: antibodies generated against tumor antigens may mistakenly target shared antigens in skin or muscle, leading to inflammation and tissue damage [5]. Cytokine release and T-cell activation may further exacerbate this autoimmune response.

Diagnosis is based on the classical Bohan and Peter criteria [6], which include proximal and symmetrical muscle weakness, characteristic cutaneous signs (heliotrope rash and Gottron’s papules), elevated serum muscle enzymes (particularly creatine kinase), electromyographic abnormalities, and muscle biopsy findings consistent with inflammatory myopathy. However, in amyopathic forms, the diagnosis relies exclusively on skin manifestations in the absence of muscle involvement [7].

Management of paraneoplastic ADM primarily involves treating the underlying malignancy [8]. Corticosteroids remain the first-line treatment for skin symptoms [9]. Hydroxychloroquine, a steroid-sparing agent, has also demonstrated efficacy in up to 80% of cases [10]. The overall prognosis is largely determined by the nature and progression of the associated malignancy [10].

In our case, the patient received a combination of CDK4/6 inhibitors and aromatase inhibitors for metastatic breast cancer, along with corticosteroids and hydroxychloroquine for skin involvement. Unfortunately, she later developed a severe COVID-19 infection. The immunosuppressed state, resulting from both cancer and treatment, likely contributed to the rapid clinical deterioration. The presence of SARS-CoV-2 infection further complicated the course, underlining the importance of monitoring infectious risk in immunocompromised patients. This case highlights how COVID-19 can severely worsen outcomes in fragile oncology patients, particularly those receiving immunosuppressive therapy.

## Conclusions

Dermatomyositis is a rare idiopathic inflammatory myopathy that primarily affects skeletal muscle and skin, often presenting with well-characterized cutaneous signs. In some cases, it may occur as a paraneoplastic manifestation of an underlying malignancy. The amyopathic form accounts for less than 20% of all dermatomyositis cases and is frequently associated with breast cancer. Given this association, clinicians should maintain a high index of suspicion and perform thorough malignancy screening in patients diagnosed with ADM.
